# C57BL/6 and A/J Mice Have Different Inflammatory Response and Liver Lipid Profile in Experimental Alcoholic Liver Disease

**DOI:** 10.1155/2015/491641

**Published:** 2015-09-13

**Authors:** Lorena Bavia, Íris Arantes de Castro, Lourdes Isaac

**Affiliations:** ^1^Departamento de Imunologia, Instituto de Ciências Biomédicas, Universidade de São Paulo, 05508-900 São Paulo, SP, Brazil; ^2^Instituto Carlos Chagas, Fundação Oswaldo Cruz, 81350-010 Curitiba, PR, Brazil

## Abstract

Alcoholic liver disease (ALD) is an important worldwide public health issue characterized by liver steatosis, inflammation, necrosis, and apoptosis of hepatocytes with eventual development of fibrosis and cirrhosis. Comparison of murine models with different inflammatory responses for ALD is important for an evaluation of the importance of genetic background in the interpretation of ethanol-induced phenotypes. Here, we investigated the role of inflammation and genetic background for the establishment of ALD using two different mouse strains: C57BL/6 (B6) and A/J. B6 and A/J mice were treated with a high fat diet containing ethanol (HFDE) and compared to the controls for 10 weeks. Hepatomegaly and steatohepatitis were similar in B6 and A/J mice, but only A/J mice were resistant to weight gain. On the other hand, HFDE-fed B6 accumulated more triglycerides (TG) and cholesterol and presented more intense cellular infiltrate in the liver when compared to HFDM-fed mice. Liver inflammatory environment was distinct in these two mouse strains. While HFDE-fed B6 produced more liver IL-12, A/J mice increased the TNF-*α* production. We concluded that mouse genetic background could dictate the intensity of the HFDE-induced liver injury.

## 1. Introduction

Alcoholic liver disease (ALD) is an important worldwide public health issue affecting millions of people every year. The first stage of liver injury is steatosis, an abnormal retention of lipids within the hepatocytes observed as a consequence of acute or chronic ingestion of ethanol, followed by reduced *β*-oxidation of fatty acids, increased triglyceride (TG) synthesis, and extrahepatic fatty acid mobilization [1]. In addition to steatosis, ALD are characterized by inflammation, necrosis, and apoptosis of hepatocytes, with eventual development of fibrosis and cirrhosis [2, 3].

The development and progression of ALD depends on the participation of Kupffer cells, Toll-like receptors (TLR), and proinflammatory cytokines [4]. Several studies have shown that activation of TLR-4 by lipopolysaccharide (LPS) triggers oxidative stress by Kupffer cells and consequent accumulation of hepatic lipids, development of inflammation, and necrosis in murine models of chronic exposure to ethanol [5–7]. Furthermore, it has been shown that chronic ethanol facilitates the translocation of bacteria and endotoxins (such as LPS) from gastrointestinal lumen into the intestinal epithelium tissue and circulatory system, finally reaching the liver where they activate Kupffer cells [8]. This interaction leads to the production of various inflammatory factors such as reactive oxygen species and cytokines, including tumor necrosis factor (TNF-) *α*, a proinflammatory cytokine, which may cause injury to hepatocytes [9]. TNF-*α* receptor knockout mice or rats treated with anti-TNF-*α* presented reduced alcohol-induced liver steatosis induced by alcohol [10].

Since ALD is clinically relevant, the search for a suitable animal model is quite pertinent to understand its etiopathogenesis [11, 12]. Treatment with high fat diets containing ethanol has been employed by many research groups to establish liver steatosis in experimental models of ALD [13, 14]. In this work, we studied the consequences of a chronic use of ethanol combined with a high fat diet in two different isogenic mouse strains C57BL/6 and A/J, commonly used in biomedical research. In addition to the different genetic backgrounds, these two mouse strains exhibit distinct inflammatory responses [15], complement system activation (A/J mice are C5-deficient [16]), drug metabolism [17], different* ad libitum *ethanol consumption [18], and weight gain [19, 20]. In this paper we explored the differences between B6 and A/J mice in ALD model to induce liver inflammation and lipid accumulation triggered by high fat diet containing ethanol.

## 2. Material and Methods

### 2.1. Animals and Ethics Statement

A/J, spontaneously C5-deficient [16], and C57BL/6 (B6) mice were maintained at the Animal Care Unit of the Institute of Biomedical Sciences, University of São Paulo. Eight- to ten-week-old male mice were used in all experiments (*n* ≥ 5). All procedures were previously approved by the Institutional Animal Ethics Committee (CEUA #057 and #086). All mice were anaesthetized with ketamine and xylazine (100 mg/kg and 10 mg/kg, resp., i.p.) before being euthanized.

### 2.2. High Fat Diets

Mice were kept in micro isolator cages (2 mice/cage) and fed with a high fat diet (HFD) containing ethanol (HFDE) as the experimental group, HFD containing maltodextrin (HFDM) as the equicaloric control or HFD alone as basal control groups. The semisolid HFD formula (Table 1) manufactured by Rhoster (Indústria e Comércio Ltda, Araçoiaba da Serra, SP, Brazil) was based on the Lieber De Carli liquid diet [21, 22], with addition of 3 g/L agar to the original composition based on an agar-gel diet as proposed by Bykov et al. [21] (http://www.rhoster.com.br/produtos/). HFDE contains 1.0 Kcal/mL of which 35% is derived from fat, 11% from carbohydrates, 18% from proteins, and 36% from ethanol (final concentration 5.3% v/v). In the HFDM composition the ethanol was replaced by maltodextrin (77.1 g/L). In the HFD, no ethanol or maltodextrin was added. Animals were fed* ad libitum *with different diets for 6 and 10 weeks before analysis. All mice received fresh diet daily. The diet consumption was monitored over 10 weeks and no significant difference was found in consumption, neither between the diets nor between the mouse strains. The mean consumption per week for B6 mice was HFD: 9.0 ± 0.79 g, HFDM: 8.2 ± 0.24 g, and HFDE: 7.5 ± 0.48 g, and for A/J mice was HFD: 8.7 ± 0.40 g, HFDM: 8 ± 0.83 g, and HFDE: 8.0 ± 0.65 g.

### 2.3. White Blood Cells Counts

Venous blood samples were harvested from orbital venous plexus with heparinized glass capillary tubes from anesthetized mice. Total white blood cell counts were measured in Neubauer chamber after diluting samples in Turk solution.

### 2.4. Histological Analysis

Liver samples were fixed in 4% buffered formalin, embedded in paraffin, sliced in 6 *μ*m sections and stained with hematoxylin/eosin (HE), and analyzed under optical microscopy at 20x, 40x, or 100x magnification using a Nikon Eclipse E200 microscope (Nikon Instruments Inc.). To observe the deposition of glycogen in the hepatocyte cytoplasm we stained paraffin-embedded liver sections with periodic acid Schiff (PAS). This procedure stains glycogen purple and the nuclei blue. Liver fibrosis was evaluated in polarized light microscope (at 40x magnification) after Picro Sirius red staining. All images were captured using a Nikon DXM1200C digital camera. A representative histopathological image from each treatment was selected.

Liver steatosis percentage was measured employing the Image J software (version 1.48k) (http://imagej.nih.gov/ij/). Ten random views on each slide of HE-stained sections were photographed at 40x magnification and the number of liver lipid vesicles was determined. In order to evaluate liver lobular inflammation the focus of cellular infiltrate was counted in ten random fields at 20x magnification on each slide of HE-stained sections.

### 2.5. Determination of Cytokines

Frozen livers were homogenized (0.2 g tissue/mL) in lysis buffer (50 mM Tris-HCl, pH 7.4; 1% NP-40; 0.25% sodium deoxycholate, 150 mM NaCl; 1 mM EDTA, 17.5 *μ*g/mL aprotinin; 5 *μ*g/mL bestatin, 10 *μ*g/mL leupeptin, 20 *μ*g/mL E-64, 1 mM Na_3_VO_4_, 10 mM Na_4_P_2_O_7_) [14]. One tablet of complete cocktail inhibitor (Roche, Indianapolis, Indiana, USA) was added for each 50 mL buffer. Samples were normalized with respect to the total protein concentrations using the Bio-Rad Protein Assay kit II (cat. 500-0002) before quantification of cytokines. The cytokine concentrations were measured by enzyme-linked immunosorbent assay (ELISA) using the following kits: TNF-*α* (BD OptEIA Mouse TNF-*α* ELISA Set, cat. 555268), IL-6 (BD OptEIA Mouse IL-6 ELISA Set, cat. 555240), IL-12p70 (BD OptEIA Mouse IL-12p70 ELISA Set, cat. 555256), and IL-17A (eBioscience Mouse ELISA Ready-SET-Go, cat. 88-7371-88).

### 2.6. Quantification of Liver Triglycerides and Cholesterol

Total lipid fraction from liver lysates (0.5 mL) was obtained after extraction with methanol: chloroform as previously described [21]. The concentrations of liver triglycerides (TG) and cholesterol were measured using the triglyceride reagent kit (cat. 87-2/250) from LabTest Diagnóstica S.A., Lagoa Santa, MG, Brazil, and cholesterol reagent kit (cat. K083) from Bioclin Quibasa, Química Básica Ltda, Belo Horizonte, MG, Brazil. Biocontrol N from Bioclin (cat. K073) and Calibra H (cat. K080) from LabTest were used as internal controls in all biochemical assays. The liver TG and cholesterol were expressed in mg* per* g of liver weight.

### 2.7. Statistical Analysis

To compare the levels of weight gain, liver to body weight ratio, TG, cholesterol, cytokines, and other parameters in different animal groups, we employed one-way ANOVA and Tukey as a posttest. We compared all diets: HFD (basal control), HFDM (pair-fed control), and HFDE (experimental group). Differences were considered statistically significant only when *P* < 0.05. All data were expressed as means ± SEM.

## 3. Results

In this study, we initially fed two different mouse strains (B6 and A/J) for 6 weeks with high fat diet containing ethanol (HFDE) to mimic ALD condition (see Supplementary Figures 1A–D available online at http://dx.doi.org/10.1155/2015/491641). Both mouse strains behaved similarly under different treatments (HFD, HFDM, or HFDE) regarding body parameters and liver steatosis (Supplementary Figures 1A and 1B). On the other hand, B6 mice HFDE-fed for 6 weeks presented more liver TG and cholesterol than A/J mice (Supplementary Figure 1C). No differences in cytokine (TNF-*α*, IL-6, IL12p70, and IL-17) concentrations were observed in the liver of both strains (Supplementary Figure 1D). Since at 6 weeks of treatment no significant differences between B6 and A/J mouse strains were observed for the majority of parameters evaluated (except for liver TG), we decided to extend the HFDE treatment for a longer period of time (10 weeks). All the data described below corresponds to this 10-week treatment period and is compared with that obtained for animal controls fed with a basal diet lacking ethanol (HFD) or with HFD containing maltodextrin (HFDM) as an equicaloric control.

### 3.1. Body Weight Gain and Hepatomegaly Development

**Figure 1:**
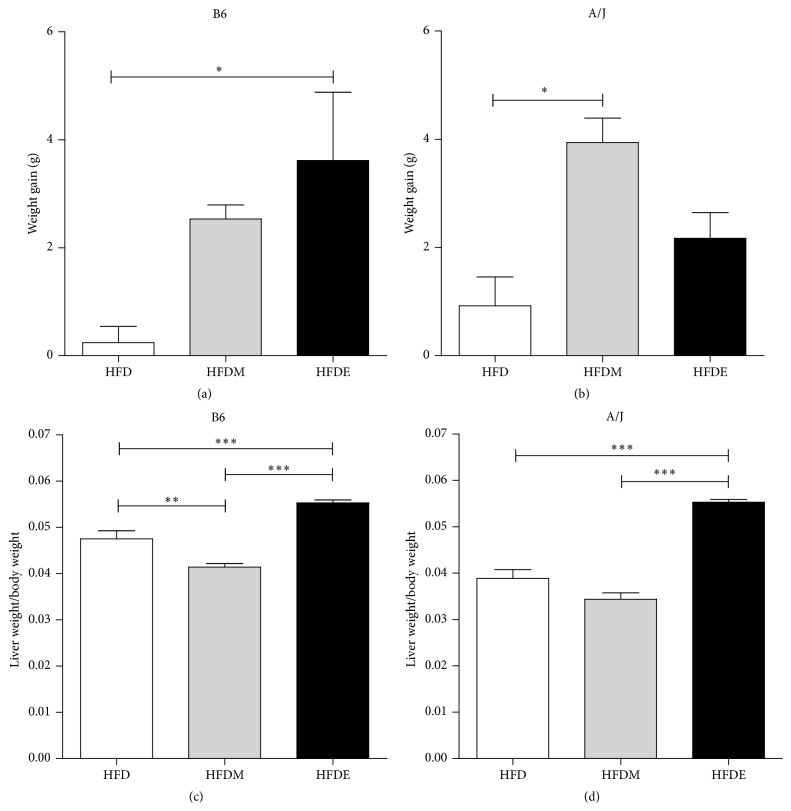
Body weight gain and development of hepatomegaly in B6 and A/J mice fed high fat diets containing ethanol. Before and after 10 weeks of treatment, B6 (a) and A/J (b) mice were weighed and the differences between initial and final weight were expressed in grams (g). Hepatomegaly was evaluated considering the liver weight/body weight ratio in B6 (c) and A/J (d) mice. Results from both B6 and A/J mice (*n* ≥ 5) were evaluated by ANOVA one-way and Tukey as a posttest. Values represent means and standard error. The differences are represented by ^*∗*^ *P* < 0.05, ^*∗∗*^ *P* < 0.01, and ^*∗∗∗*^ *P* < 0.001. HFD: high fat diet, HFDM: HFD containing maltodextrin, HFDE: HFD containing ethanol.

Ten weeks after feeding with all diets we evaluated weight gain and liver enlargement. Figures 1(a) and 1(b) show that A/J mice were more resistant to gain weight when HFDE-fed than B6 mice. Figures 1(c) and 1(d) show that both mouse strains developed hepatomegaly (increase in liver weight/body weight ratio) after feeding with HFDE while no hepatomegaly was observed after HFD and HFDM treatments. Under normal chow feeding B6 and A/J mice (8 weeks old) present 0.033 and 0.029 liver weight/body weight ratio, respectively.

### 3.2. Liver Histopathological Alterations

**Figure 2:**
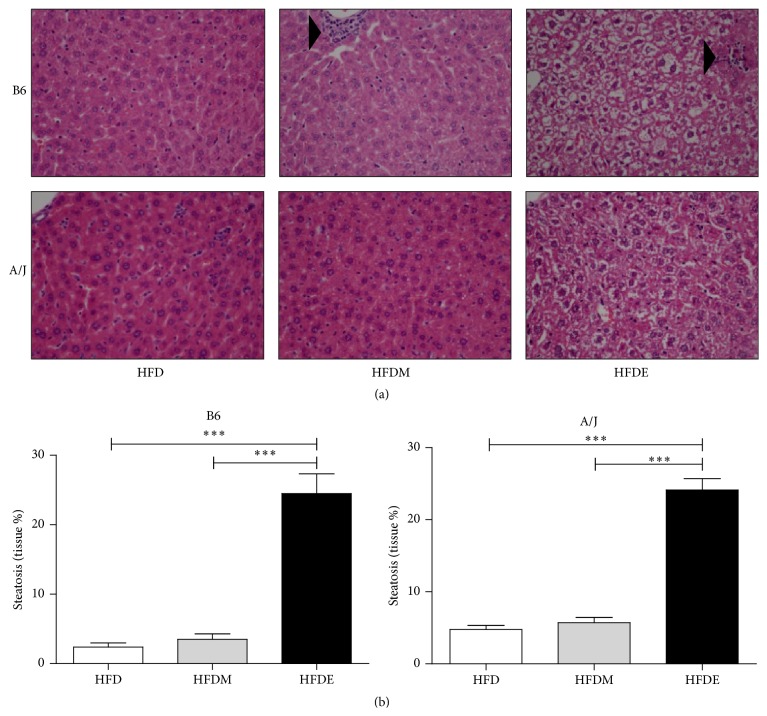
Liver alterations in B6 and A/J mice supplemented with ethanol. Liver sections were obtained from B6 and A/J mice fed with HFD, HFDM, or HFDE for 10 weeks. Liver sections were formalin-fixed and paraffin-embedded before HE staining. Arrowheads point to inflammatory cell infiltrates (magnification 40x) (a). Ten random fields of each slide at magnification of 40x were photographed and the percentage of steatosis was measured using the Image J program (b). Eight- to ten-week-old male mice were used in all experiments (*n* ≥ 5). HFD: high fat diet, HFDM: HFD containing maltodextrin, HFDE: HFD containing ethanol.

Morphological alterations in the livers of B6 and A/J mice were evaluated by histopathology analysis (Figure 2(a)) after 10 weeks of treatment with all three high fat diets (HFD, HFDM, and HFDE). Liver from HFDE-fed B6 mice exhibited more focus of cellular infiltrate (3.1 ± 0.7) when compared to HFDM-fed mice (1.2 ± 0.2) and HFD-fed mice (2.5 ± 0.5), as represented in Supplementary Figure 2A. On the other hand, HFDE- and HFDM-fed A/J mice presented increased focus of cellular infiltrate in the liver, 6.3 ± 4.4 and 5.2 ± 2.5, respectively, when compared to HFD counting, 2.5 ± 0.3 (Supplementary Figure 2B). When liver sections were stained using Picro Sirius red to identify the presence of type I (constitutively expressed) and III (expressed during tissue repair) collagen fibers, no differences were observed in these two HFDE-fed mouse strains, suggesting that livers from B6 and A/J mice apparently repair hepatic tissue damage in a similar manner (Supplementary Figure 3). However, it is interesting to note that HFD- and HFDM-fed A/J mice presented a different pattern of collagen fibers when compared to B6 mice, corroborating with the focus of cellular infiltrate counting results. These results suggest that B6 and A/J mice may have different inflammatory responses in liver diseases depending on the mouse genetic background and on the stimulus used to trigger inflammation.

Although morphologically liver steatosis was more evident in liver from B6 than from A/J HFDE-fed mice, both mouse strains presented a similar tissue percentage of steatosis (Figure 2(b)). In order to confirm that the vesicles observed in hepatic histology comprised steatosis and not glycogen accumulation, we performed a PAS staining protocol. In agreement with the observed hepatomegaly (Figure 1) and steatosis (Figure 2), deposition of glycogen was similar in hepatocytes from both mouse strains (Supplementary Figure 4). Even though deposition of glycogen was observed in both strains, most vesicles observed in liver histopathology represent lipid accumulation. No significant differences in serum activities of the liver alanine and aspartate transferases (ALT and AST) were detected in both mouse strains when treated with HFD, HFDM, or HFDE (data not shown).

**Figure 3:**
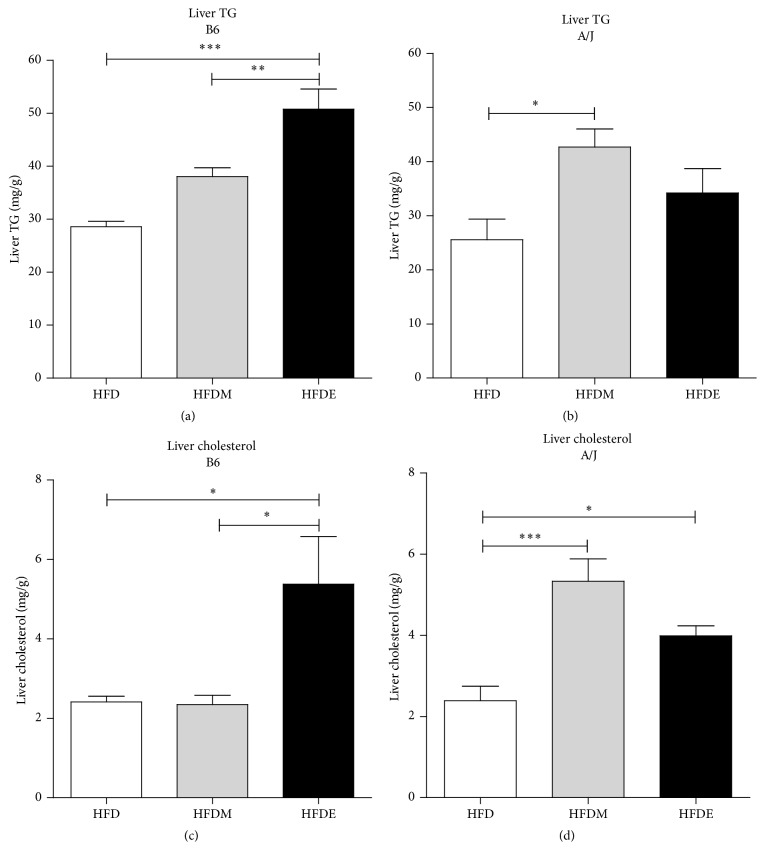
Triglycerides and cholesterol contents in the liver. B6 and A/J mice were fed with HFD, HFDM, and HFDE for 10 weeks. The liver was harvested, the total lipid was extracted, and the liver triglycerides ((a) and (b)) and liver cholesterol ((c) and (d)) were measured. Results from both B6 and A/J mice (*n* ≥ 5) were evaluated by ANOVA one-way and Tukey as a posttest. Values represent means and standard error. The differences are represented by ^*∗*^ *P* < 0.05, ^*∗∗*^ *P* < 0.01, and ^*∗∗∗*^ *P* < 0.001. TG: triglycerides, HFD: high fat diet, HFDM: HFD containing maltodextrin, HFDE: HFD containing ethanol.

To further explore the steatohepatitis observed in B6 and A/J mice after HFDE (Figure 2), we measured their liver TG and cholesterol concentrations. After treatment with ethanol (HFDE), only B6 mice presented increased liver TG and cholesterol concentrations when compared to the HFD and HFDM control groups (Figure 3).

Taken together, the above results demonstrated that, in this ALD model, B6 mice accumulate more TG and cholesterol in the liver HFDE-induced. In addition, B6 mice exhibited more hepatic inflammatory infiltrate than A/J mice when HFDE-fed group was compared with HFDM control.

### 3.3. Blood Leukocyte Profile and Liver Inflammatory Environment

**Figure 4:**
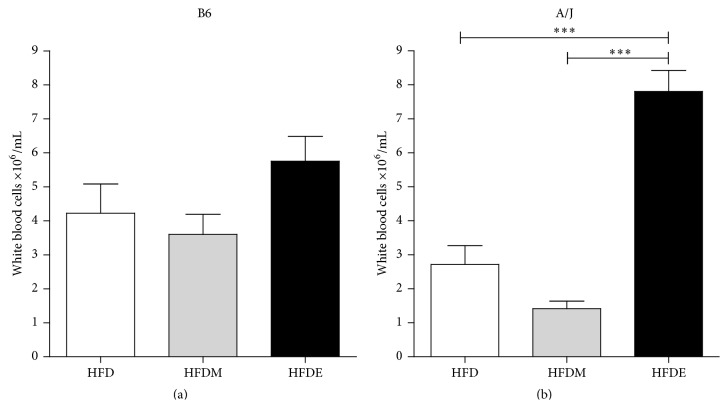
Total number of circulating leukocytes. B6 (a) and A/J (b) mice were fed with HFD, HFDM, or HFDE for 10 weeks. Results from both B6 and A/J mice (*n* ≥ 5) were evaluated by ANOVA one-way and Tukey as a posttest. Values represent means and standard error. The differences are represented by ^*∗*^ *P* < 0.05, ^*∗∗*^ *P* < 0.01, and ^*∗∗∗*^ *P* < 0.001. HFD: high fat diet, HFDM: HFD containing maltodextrin, HFDE: HFD containing ethanol.

To monitor the inflammatory environment, the number of circulating leukocytes was measured in the two mouse strains. No differences were observed in B6 mice (Figure 4(a)) treated with different diets. However, HFDE-fed A/J mice showed a significant increase in total number of leukocytes when compared to the HFD and/or HFDM control groups (Figure 4(b)). The basal levels of blood leukocytes in B6 and A/J mice (8 weeks old) are 8.3 ± 2.6 × 10^6^ cells/mL and 2.6 ± 1 × 10^6^ cells/mL, respectively, when they were fed with regular chow.

**Figure 5:**
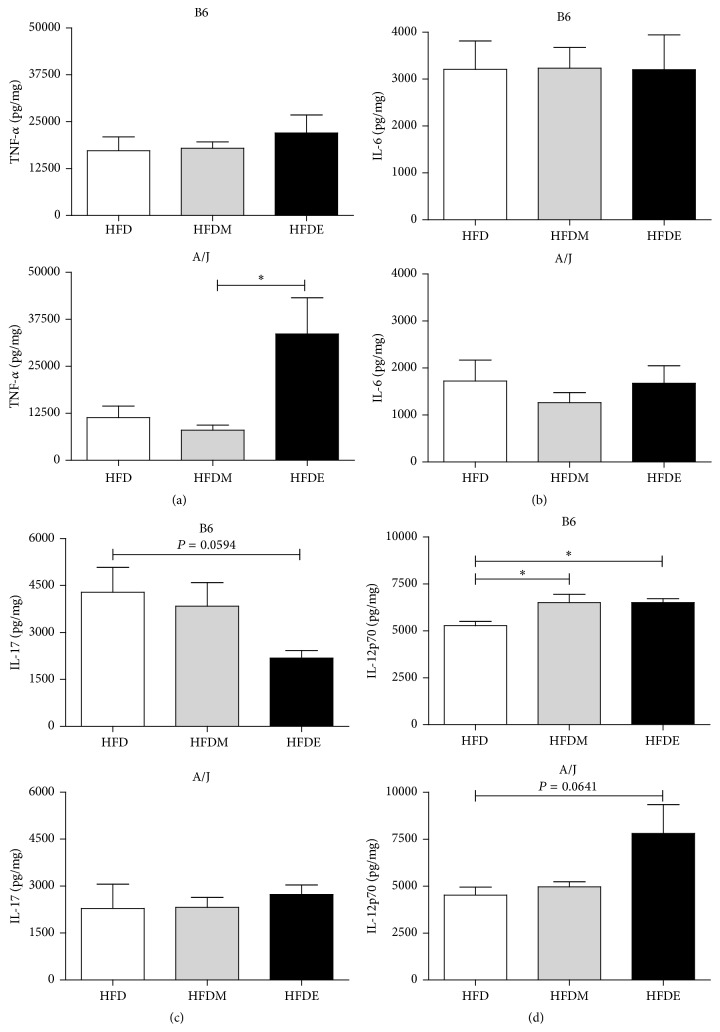
Liver cytokines in B6 and A/J mice after ethanol feeding for 10 weeks. Liver TNF-*α* (a), IL-6 (b), IL-17A (c), and IL-12p70 (d) concentrations in B6 and A/J mice after 10 weeks of HFD, HFDM, and HFDE. Livers were collected and frozen in liquid nitrogen. Lysates were prepared from frozen samples and cytokines (pg/mg protein) were measured by ELISA. Results from both B6 and A/J mice (*n* ≥ 5) were evaluated by ANOVA one-way and Tukey as a posttest. Values represent means and standard error. The differences are represented by ^*∗*^ *P* < 0.05, ^*∗∗*^ *P* < 0.01, and ^*∗∗∗*^ *P* < 0.001. HFD: high fat diet, HFDM: HFD containing maltodextrin, HFDE: HFD containing ethanol.

The hepatic environment becomes highly inflammatory under ALD [9]. Considering that B6 and A/J mice behaved quite distinctly, we measured the concentrations of important cytokines (TNF-*α*, IL-6, IL-12p70, and IL-17) in the liver of both mice under the different treatments (Figure 5). No statistically significant differences in liver TNF-*α* level were observed for the B6 mouse groups. On the other hand, liver TNF-*α* levels in HFDE-fed A/J mice were significantly increased when compared to the HFDM control (Figure 5(a)). The treatment with HFDE did not affect the liver production of IL-6 and IL-17 when compared to HFDM in both mouse strains (Figures 5(b) and 5(c)). However, there was a significant increase in IL-12p70 levels in HFDE and HFDM-fed B6 mice when compared to the HFD group (Figure 5(d)).

The hepatic changes induced by the consumption of HFDE in both mouse strains are summarized in Table 2: (i) development of hepatomegaly was similar in both mouse strains; (ii) B6 accumulated more TG and cholesterol in the liver than A/J; (iii) circulating leukocytes are present in higher counting in A/J mice blood when compared to B6; (iv) liver proinflammatory environment involved in steatosis is different in B6 and A/J: HFDE-fed B6 mice produce higher levels of liver IL-12p70 while HFDE-fed A/J mice release more liver TNF-*α* cytokine.

## 4. Discussion

Understanding the mechanisms involved in the progression of liver steatosis in ALD is essential to design new therapeutic strategies. The pathogenesis of ALD is complex and not fully understood [23] and so far not many experimental animal models have explored the influence of the inflammatory environment for disease progression. In order to investigate the contribution of inflammatory response and genetic background, we used B6 and A/J mice, two strains commonly used in the laboratory but with quite different cytokine profiles, blood cell counts, and metabolism of alcohol and lipids [15, 17, 18, 20, 24]. Moreover, while B6 mice carry normal complement-mediated activity, A/J is C5-deficient [16].

Similar to what is observed in human patients, A/J mice are resistant to gain weight when HFDE-fed and both mice develop hepatomegaly. Liver enlargement occurs as a consequence of lipid accumulation, which is induced by the chronic ethanol consumption together with the high fat diet [1, 21, 22]. We observed fatty liver in both mouse strains subjected to the HFDE diet, in agreement with other previously reported experimental models of alcoholism [14, 25]. While liver steatosis was observed in both HDFE-fed mouse strains, liver TG and cholesterol levels increased only in B6 mice. The resistance of A/J mice to accumulate TG has been previously observed by Kondo et al. [26] and Surwit et al. [27], where a distinct regulation of genes linked to lipid metabolism (carnitine palmitoyltransferase I, liver fatty acid binding protein, pyruvate dehydrogenase kinase-4, and NADP^+^-dependent cytosolic malic enzyme) was reported in A/J mice. This mouse strain is considered obesity-resistant and upregulates genes related with lipid metabolism in the small intestine [26]. On the other hand, B6 mice are considered obesity-prone with downregulating the expression of the same genes, suggesting that lipid metabolism in the small intestine and genetic background are associated with susceptibility to obesity. Likewise, in a diabetes type II induced model [27], A/J mice were observed to be resistant while B6 mice were prone to development disease. It is noteworthy that A/J mice are deficient in complement C5 component [16], which suggests that this protein may play a role in lipid metabolism in the liver. In agreement with this hypothesis, C5-deficient mice (B10.D2/oSnJ) do not accumulate TG in liver in an ALD model to the same extent as that observed in congenic C5-sufficient mice (B10.D2/nSnJ) [14]. TG accumulation within hepatocytes is lipotoxic and may represent an early step in the etiopathogenesis of ALD [28] and the nature of factors related to this lipid deposition remains to be fully understood. It is well known that the component C5, especially its fragment C5a and its receptor C5aR, has a central role in the inflammatory response [16, 29–32]. In addition, the alternative C5a receptor, C5L2, which can also bind C3a and C3a_desarg_, is associated with TG synthesis and glucose capture by adipocytes [33]. Therefore, C5aR and C5L2 may act synergically under high fat dietary and inflammatory conditions.

As described above, innate immunity plays an important role in the development of ALD [4, 8, 14] and the proinflammatory cytokines TNF-*α* and IL-6 contribute to the maintenance of chronic inflammation observed in ALD [34–37]. TNF-*α* levels are higher in serum [36–38] and monocytes [39] from patients with ALD and in serum [40] and liver [10] in murine ALD models. Liver TNF-*α* is induced at the initial stages of the disease and is strongly correlated with development of ethanol-induced liver injury [8]. Therefore, the local production of cytokines in the liver of B6 and A/J mice was measured. Interestingly, we observed that only A/J mice presented increased liver TNF-*α* levels when HDFE-fed for 10 weeks. Even in the lack of component C5 A/J mice HFDE-fed were able to produce and release TNF-*α* by macrophages (as Kupffer cells) in the liver. Translocation of bacteria and endotoxins (such as LPS) from gastrointestinal lumen to circulatory system is observed during ALD development. Consequently, TLR-4 present in Kupffer cells can be triggered by LPS and produce TNF-*α* [6, 8]. Probably, the stimulus to secrete liver TNF-*α* in A/J mice fed with HFDE may be from TLR-4 activation in resident cells.

The cytokine IL-6 has two important functions in the inflammatory response during ALD: (i) to stimulate proinflammatory cytokines production by macrophages and (ii) to protect the liver recovering from necrosis induced by inflammation [41]. We observed that B6 mice presented twice the level of liver IL-6 compared to A/J mice, independent of the diet. Similar results were observed in previous work from our group where we evaluated the acute ethanol-induced inflammatory response [24]. Moreover, in the acute model, A/J mice were more susceptible to ethanol-induced liver damage than B6 mice [24]. In another study, Roychowdhury et al. found that liver IL-6 levels increased at 3, 21, and 40 days in a murine model of ALD [8]. Taken together, our results suggest the importance of IL-6 at the early stages (3 days to 5 weeks), and not in late stages (10 weeks), of ALD and corroborate the conclusions arrived at by other groups [8, 42].

Another cytokine evaluated in the present study was IL-12p70, which is involved in initiation of the cell-mediated immune response. IL-12 is responsible for differentiation of T cells to Th1 cells profile. Although there is no work evaluating liver IL-12 levels in murine models of ALD, this cytokine has been suggested as a serum biomarker of status of continuous alcohol consumption in humans and serum IL-12 levels reflect the different stages of alcoholic liver disease in patients [43]. We observed an increase in liver IL-12p70 in both HFDE-fed B6 and A/J mice. The IL-12 increases in HFDM- and HFDE-fed B6 mice suggest a synergic effect of HFD and maltodextrin or ethanol towards IL-12 production. This is the first report of an increase of the liver IL-12 in a murine model of ALD. The ethanol-induced liver IL-12 enhancement reinforces the role of cell-mediated immune response in liver steatosis.

Recent studies show that the IL-17^+^ cells and high levels of IL-17 cytokine are present in ALD patients [44, 45]. In addition, a correlation between IL-17 secretion by liver infiltrated cells and the severity of liver fibrosis was observed in these patients [45]. Although IL-17 is a cytokine produced by lymphocytes from acquired immunity it can also contribute to the recruitment of monocytes, neutrophils, and leukocytes to the inflammatory site [46]. In contrast to what is observed in ALD patients, our results showed a decrease of liver IL-17 in HFDE-fed B6 mice when compared with the HFD basal control group. Furthermore, liver IL-6 and liver IL-17 levels in B6 mice are 2-fold greater than those observed in the A/J.

Immunopathological characteristics were observed in our ALD model. Liver steatosis in ALD was observed during treatment with HFDE in both B6 and A/J mice. It is evident that genetic background and the inflammatory response are important factors in determining how each mouse strain develops and maintains liver steatosis under a chronic inflammatory environment. The different inflammatory response developed for each mouse strain could result in the different pathologies and lipid accumulations observed. It is possible that in HFDE-fed A/J mice the inflammatory response (probably innate immunity-mediated chronic inflammation) contributes to the maintenance of steatosis but protects this strain from liver TG and cholesterol accumulation. Due to lack of C5 in A/J mice [16] the activation of the inflammatory response could develop more slowly. Considering that 10 weeks of ethanol feeding revealed remarkable differences between B6 and A/J mice, the physiological basis of the late stages of ALD differences should be explored.

## 5. Conclusion

Our ALD model presented pathological alterations in the liver of both mouse strains after 10 weeks of feeding. These symptoms are similar to those observed in human alcoholic patients. Both B6 and A/J mouse strains develop similar ALD steatosis but they exhibit distinct inflammatory phenotypes and lipid accumulation in the liver: B6 mice produce less cytokines involved in innate immunity but they accumulate more TG and cholesterol in the liver when compared to A/J mice under the same treatment. Whether the differences observed in B6 and A/J mouse strains are exclusively attributed to multiple differences in genetic background or whether the complement protein C5 plays a specific role in maintenance of liver inflammatory environment and lipid metabolism in this pathological state remains to be investigated.

## Supplementary Material

B6 and A/J mice were randomly divided in three groups and fed with HFD, or HFDM or HFDE for 6 weeks. Body parameters (A), liver steatosis (B), liver lipid composition (C) and liver inflammation (D) were evaluated in order to highlight ethanol-induced differences between B6 and A/J mice. Although, B6 and A/J mice had difficult to gain body weight both mice had increased liver to body weight ratio when HFDE-fed (A). In addition, HFDE-induced liver steatosis percentage was similar between B6 and A/J mice (B). Nonetheless, HFDE-fed A/J mice were resistant in accumulates liver TG and cholesterol when compared with B6 mice (C). Finally, liver inflammation was investigated through TNF-*α*, IL-6, IL-12p70 and IL-17 cytokines levels. No significant differences between the diets groups were found. These cytokines were chosen because: TNF-*α* is an important inflammatory marker in ALD patients and mice models of ALD; IL-6 is a potent inflammatory mediator of the acute phase response; IL-12p70 is involved in differentiation of naive T into Th1 cells and stimulates TNF-*α* production; IL-17 mediates inflammatory response and recruits cells to inflammation site.

## Figures and Tables

**Table 1:** High fat diet composition.

Ingredient	g/L of diet
Casein (100 mesh)	41.4
L-Cystine	0.5
DL-methionine	0.3
Corn oil	8.5
Olive oil	28.4
Safflower oil	2.7
Maltodextrin	25.6
Cellulose	10.0
Salt mix^*∗*^	8.75
Vitamin mix^*∗∗*^	2.5
Choline bitartrate	0.53
Guar gum^$^	3.0
Agar^&^	3.0

^*∗*^Salt mix (g/kg): calcium phosphate, dibasic (500 g), sodium chloride (74 g), potassium citrate, monohydrate (220 g), potassium sulfate (52 g), magnesium oxide (24 g), manganous sulfate H_2_O (4.6 g), ferrous sulfate 7 H_2_O (4.95 g), zinc carbonate (1.6 g), cupric carbonate (0.3 g), potassium iodate (0.01 g), sodium selenite (0.01 g), chromium potassium sulfate (0.55 g), sodium fluoride (0.06 g), sucrose, finely powdered (117.92 g).

^*∗∗*^Vitamin mix (g/kg): thiamin-HCl (0.6 g), riboflavin (0.6 g), pyridoxine-HCl (0.7 g), niacin (3.0 g), calcium pantothenate (1.6 g), folic acid (0.2 g), biotin (0.02 g), vitamin B12 (10.00 g), vitamin A acetate, 500,000 IU/g (4.8 g), vitamin D3, 400,000 IU/g (0.4 g), vitamin E acetate, 500 IU/g (24 g), menadione sodium bisulfite (0.08 g), p-amino benzoic acid (5.0 g), inositol (10 g), and dextrose (939 g).

^$^Replace the Xanthan gum from original diet.

^&^Adapted from Bykov et al. [21].

**Table 2:** Summary of main results found at 10th week after B6 and A/J fed with HFDE compared with HFDM.

Diet mice	HFDE versus HFDM	
	B6	A/J
Hepatomegaly	↑	↑
Steatosis	↑	↑
TG	↑	
Cholesterol	↑	
Blood leukocytes		↑
Liver TNF-*α*		↑

↓: decrease.

↑: increase.

## References

[1] Lieber C. S. (2004). Alcoholic fatty liver: its pathogenesis and mechanism of progression to inflammation and fibrosis. *Alcohol*.

[2] Wang H. J., Gao B., Zakhari S., Nagy L. E. (2012). Inflammation in alcoholic liver disease. *Annual Review of Nutrition*.

[3] Cohen J. I., Nagy L. E. (2011). Pathogenesis of alcoholic liver disease: interactions between parenchymal and non-parenchymal cells. *Journal of Digestive Diseases*.

[4] Nagy L. E. (2003). Recent insights into the role of the innate immune system in the development of alcoholic liver disease. *Experimental Biology and Medicine*.

[5] Hines I. N., Wheeler M. D. (2004). Recent advances in alcoholic liver disease III. Role of the innate immune response in alcoholic hepatitis. *American Journal of Physiology: Gastrointestinal and Liver Physiology*.

[6] Roh Y. S., Seki E. (2013). Toll-like receptors in alcoholic liver disease, non-alcoholic steatohepatitis and carcinogenesis. *Journal of Gastroenterology and Hepatology*.

[7] Hritz I., Mandrekar P., Velayudham A. (2008). The critical role of toll-like receptor (TLR) 4 in alcoholic liver disease is independent of the common TLR adapter MyD88. *Hepatology*.

[8] Roychowdhury S., McMullen M. R., Pritchard M. T. (2009). An early complement-dependent and TLR-4-independent phase in the pathogenesis of ethanol-induced liver injury in mice. *Hepatology*.

[9] Nagata K., Suzuki H., Sakaguchi S. (2007). Common pathogenic mechanism in development progression of liver injury caused by non-alcoholic or alcoholic steatohepatitis. *The Journal of Toxicological Sciences*.

[10] Iimuro Y., Gallucci R. M., Luster M., Kono H., Thurman R. G. (1997). Antibodies to tumor necrosis factor alfa attenuate hepatic necrosis and inflammation caused by chronic exposure to ethanol in the rat. *Hepatology*.

[11] Mathews S., Xu M., Wang H., Bertola A., Gao B. (2014). Animals models of gastrointestinal and liver diseases. Animal models of alcohol-induced liver disease: pathophysiology, translational relevance, and challenges. *The American Journal of Physiology—Gastrointestinal and Liver Physiology*.

[12] Brandon-Warner E., Schrum L. W., Schmidt C. M., McKillop I. H. (2012). Rodent models of alcoholic liver disease: of mice and men. *Alcohol*.

[13] Cohen J. I., Roychowdhury S., McMullen M. R., Stavitsky A. B., Nagy L. E. (2010). Complement and alcoholic liver disease: role of C1q in the pathogenesis of ethanol-induced liver injury in mice. *Gastroenterology*.

[14] Pritchard M. T., McMullen M. R., Stavitsky A. B. (2007). Differential contributions of C3, C5, and decay-accelerating factor to ethanol-induced fatty liver in mice. *Gastroenterology*.

[15] Mills C. D., Kincaid K., Alt J. M., Heilman M. J., Hill A. M. (2000). M-1/M-2 macrophages and the Th1/Th2 paradigm. *The Journal of Immunology*.

[16] Wetsel R. A., Fleischer D. T., Haviland D. L. (1990). Deficiency of the murine fifth complement component (C5). A 2-base pair gene deletion in a 5′-exon. *The Journal of Biological Chemistry*.

[17] Caspi R. R. (2002). Th1 and Th2 responses in pathogenesis and regulation of experimental autoimmune uveoretinitis. *International Reviews of Immunology*.

[18] Yoneyama N., Crabbe J. C., Ford M. M., Murillo A., Finn D. A. (2008). Voluntary ethanol consumption in 22 inbred mouse strains. *Alcohol*.

[19] Gallou-Kabani C., Vigé A., Gross M.-S. (2007). C57BL/6J and A/J mice fed a high-fat diet delineate components of metabolic syndrome. *Obesity*.

[20] Fraulob J. C., Ogg-Diamantino R., Fernandes-Santos C., Aguila M. B., Mandarim-de-Lacerda C. A. (2010). A mouse model of metabolic syndrome: Insulin resistance, fatty liver and Non-Alcoholic Fatty Pancreas Disease (NAFPD) in C57BL/6 mice fed a high fat diet. *Journal of Clinical Biochemistry and Nutrition*.

[21] Bykov I., Palmén M., Piirainen L., Lindros K. O. (2004). Oral chronic ethanol administration to rodents by agar gel diet. *Alcohol and Alcoholism*.

[22] Lieber C. S., DeCarli L. M. (1982). The feeding of alcohol in liquid diets: two decades of applications and 1982 update. *Alcoholism: Clinical and Experimental Research*.

[23] Krawczyk M., Bonfrate L., Portincasa P. (2010). Nonalcoholic fatty liver disease. *Best Practice & Research: Clinical Gastroenterology*.

[24] Bavia L., Maiorka P. C., Isaac L. (2013). The influence of genetic background of C57BL/6 and A/J mice on the development of acute inflammatory response induced by alcohol. *Revista da Sociedade Brasileira de Ciências em Animais de Laboratório*.

[25] Bykov I. L., Väkevä A., Järveläinen H. A., Meri S., Lindros K. O. (2004). Protective function of complement against alcohol-induced rat liver damage. *International Immunopharmacology*.

[26] Kondo H., Minegishi Y., Komine Y. (2006). Differential regulation of intestinal lipid metabolism-related genes in obesity-resistant A/J vs. Obesity-prone C57BL/6J mice. *American Journal of Physiology: Endocrinology and Metabolism*.

[27] Surwit R. S., Seldin M. F., Kuhn C. M., Cochrane C., Feinglos M. N. (1991). Control of expression of insulin resistance and hyperglycemia by different genetic factors in diabetic C57BL/6J mice. *Diabetes*.

[28] Koteish A., Diehl A. M. (2001). Animal models of steatosis. *Seminars in Liver Disease*.

[29] Klos A., Tenner A. J., Johswich K.-O., Ager R. R., Reis E. S., Köhl J. (2009). The role of the anaphylatoxins in health and disease. *Molecular Immunology*.

[30] Guo R.-F., Ward P. A. (2005). Role of C5a in inflammatory responses. *Annual Review of Immunology*.

[31] Ember J. A., Jagels M. A., Hugli T. E., Volanakis J. E., Frank M. M. (1998). Characterization of complement anaphylatoxins and their biological responses. *The Human Complement System in Health and Disease*.

[32] Hawlisch H., Belkaid Y., Baelder R., Hildeman D., Gerard C., Köhl J. (2005). C5a negatively regulates toll-like receptor 4-induced immune responses. *Immunity*.

[33] MacLaren R., Cui W., Cianflone K. (2008). Adipokines and the immune system: an adipocentric view. *Advances in Experimental Medicine and Biology*.

[34] Shoelson S. E., Herrero L., Naaz A. (2007). Obesity, inflammation, and insulin resistance. *Gastroenterology*.

[35] Valenti L., Fracanzani A. L., Fargion S. (2009). The immunopathogenesis of alcoholic and nonalcoholic steatohepatitis: two triggers for one disease?. *Seminars in Immunopathology*.

[36] Khoruts A., Stahnke L., McClain C. J., Logan G., Allen J. I. (1991). Circulating tumor necrosis factor, interleukin-1 and interleukin-6 concentrations in chronic alcoholic patients. *Hepatology*.

[37] McClain C. J., Barve S., Barve S., Deaciuc I., Hill D. B. (1998). Tumor necrosis factor and alcoholic liver disease. *Alcoholism: Clinical and Experimental Research*.

[38] Vidali M., Hietala J., Occhino G. (2008). Immune responses against oxidative stress-derived antigens are associated with increased circulating tumor necrosis factor-alpha in heavy drinkers. *Free Radical Biology and Medicine*.

[39] McClain C. J., Cohen D. A. (1989). Increased tumor necrosis factor production by monocytes in alcoholic hepatitis. *Hepatology*.

[40] Yin M., Wheeler M. D., Kono H. (1999). Essential role of tumor necrosis factor *α* in alcohol-induced liver injury in mice. *Gastroenterology*.

[41] Miller A. M., Wang H., Bertola A. (2011). Inflammation-associated interleukin-6/signal transducer and activator of transcription 3 activation ameliorates alcoholic and nonalcoholic fatty liver diseases in interleukin-10-deficient mice. *Hepatology*.

[42] Gao B. (2012). Hepatoprotective and anti-inflammatory cytokines in alcoholic liver disease. *Journal of Gastroenterology and Hepatology*.

[43] Tung K.-H., Huang Y.-S., Yang K.-C., Perng C.-L., Lin H.-C., Lee S.-D. (2010). Serum interleukin-12 levels in alcoholic liver disease. *Journal of the Chinese Medical Association*.

[44] Lafdil F., Miller A. M., Ki S. H., Gao B. (2010). Th17 cells and their associated cytokines in liver diseases. *Cellular and Molecular Immunology*.

[45] Lemmers A., Moreno C., Gustot T. (2009). The interleukin-17 pathway is involved in human alcoholic liver disease. *Hepatology*.

[46] Meng F., Wang K., Aoyama T. (2012). Interleukin-17 signaling in inflammatory, Kupffer cells, and hepatic stellate cells exacerbates liver fibrosis in mice. *Gastroenterology*.

