# Supplementary material for: C57BL/6 and A/J Mice Have Different Inflammatory Response and Liver Lipid Profile in Experimental Alcoholic Liver Disease
Source: Mediators Inflamm. 2015 Sep 13;2015:491641. doi: 10.1155/2015/491641 (PMC4584053; doi:10.1155/2015/491641)

Supplem. Fig. 1

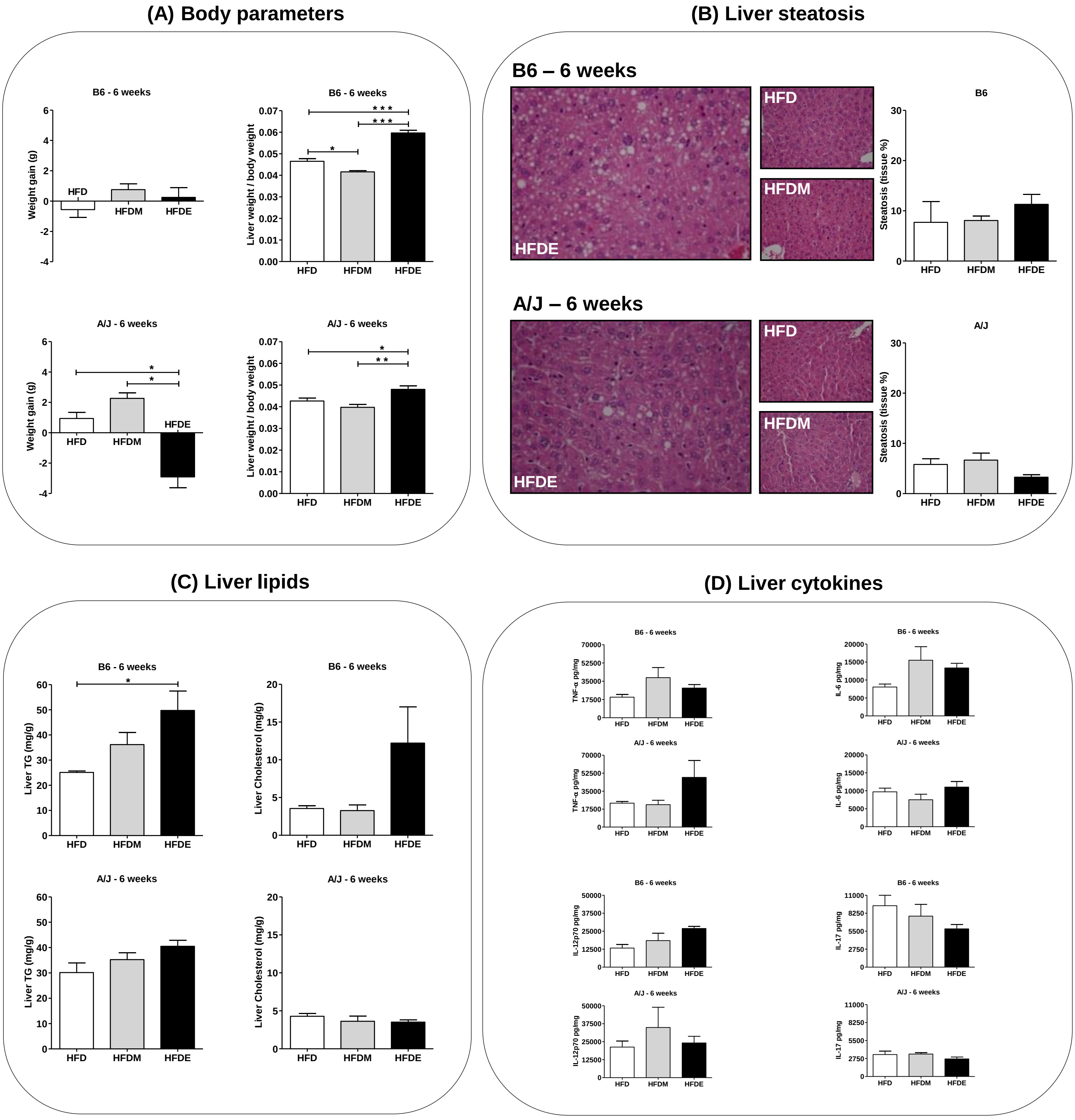

Supplem. Fig. 2

(A)

(B)

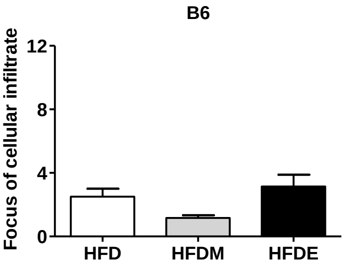

Supplem. Fig. 3

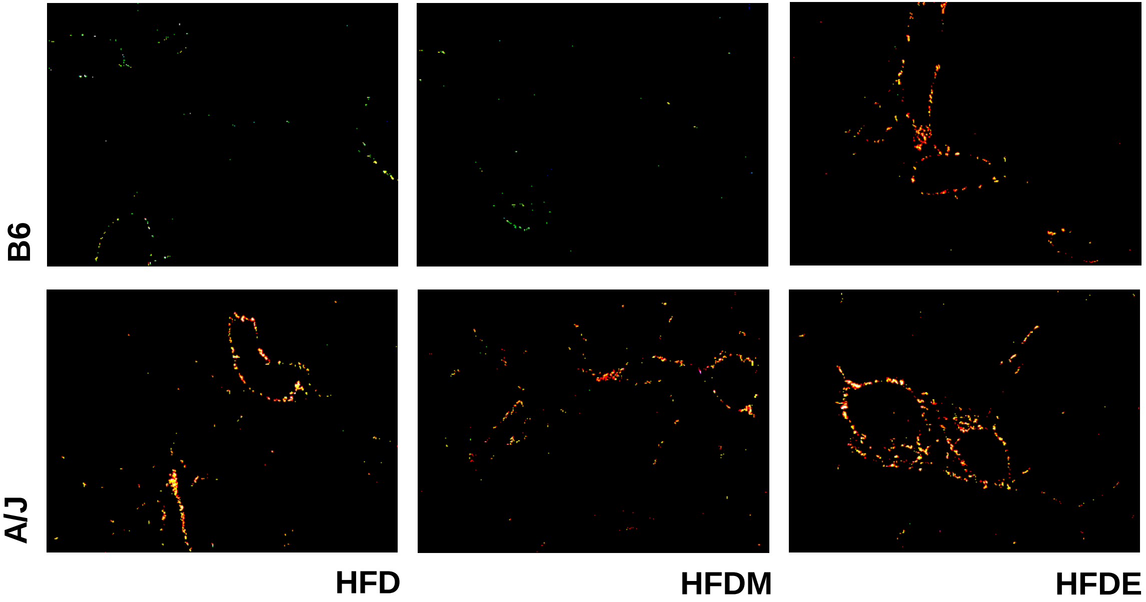

Supplem. Fig. 4

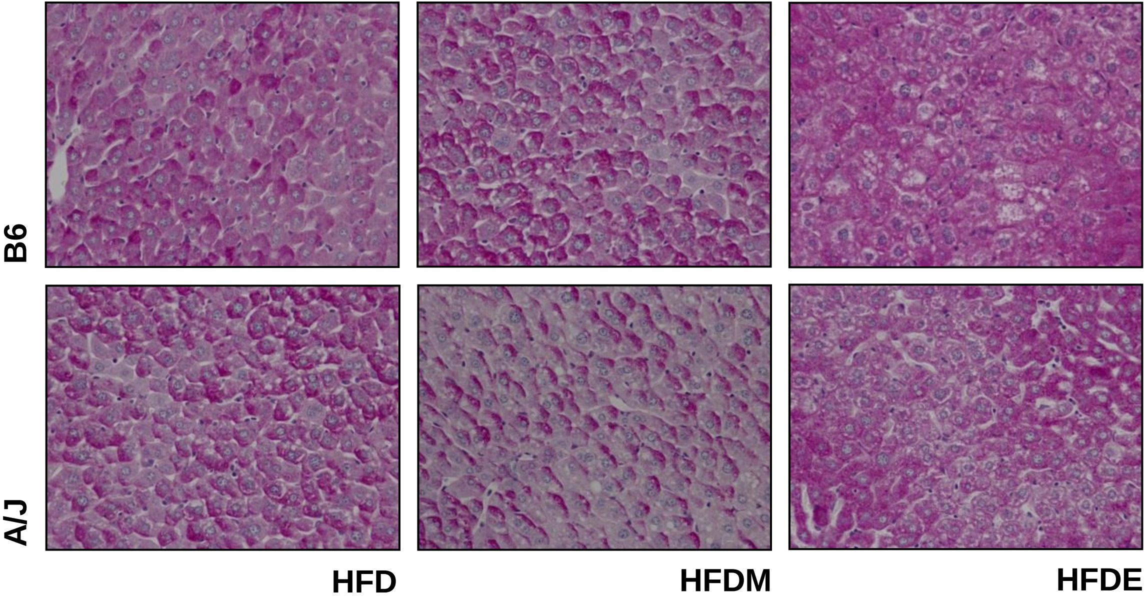

Supplement: Supplementary file 1 — B6 and A/J mice were randomly divided in three groups and fed with HFD, or HFDM or HFDE for 6 weeks. Body parameters (A), liver steatosis (B), liver lipid composition (C) and liver inflammation (D) were evaluated in order to highlight ethanol-induced differences between B6 and A/J mice. Although, B6 and A/J mice had difficult to gain body weight both mice had increased liver to body weight ratio when HFDE-fed (A). In addition, HFDE-induced liver steatosis percentage was similar between B6 and A/J mice (B). Nonetheless, HFDE-fed A/J mice were resistant in accumulates liver TG and cholesterol when compared with B6 mice (C). Finally, liver inflammation was investigated through TNF-α, IL-6, IL-12p70 and IL-17 cytokines levels. No significant differences between the diets groups were found. These cytokines were chosen because: TNF-α is an important inflammatory marker in ALD patients and mice models of ALD; IL-6 is a potent inflammatory mediator of the acute phase response; IL-12p70 is involved in differentiation of naive T into Th1 cells and stimulates TNF-α production; IL-17 mediates inflammatory response and recruits cells to inflammation site. [file 491641.f1.pdf]
